# Apoptosis-related microRNA changes in the right atrium induced by remote ischemic perconditioning during valve replacement surgery

**DOI:** 10.1038/srep18959

**Published:** 2016-01-07

**Authors:** Qinghua Hu, Wanjun Luo, Lingjin Huang, Rimao Huang, Ri Chen

**Affiliations:** 1 Department of cardiovascular surgery, Xiangya Hospital, Central-South University, Changsha, Hunan, China, 410078

## Abstract

We previously found that remote ischemic perconditioning (RIPerc) was effective in attenuating myocardial injury during cardiac surgery. Given that microRNAs (miRs) act as an important player in ischemic/reperfusion (I/R) injury and apoptosis, this study aimed to investigate whether RIPerc reduces apoptosis in atrial myocardium and which apoptosis-related miRs are involved during valve replacement surgery. Here, we demonstrated that RIPerc inhibited apoptosis in atrial myocardium during cardiac ischemia and that 17 miRs showed at least a 1.5-fold change in expression after ischemia. Of the 17 miRs, 9 miRs, including miR-1, miR-21, miR-24, and miR-195, which are related to apoptosis, exhibited different expression patterns in the RIPerc group compared with the control. Using qRT-PCR and Western blotting, we demonstrated that miR-1 and miR-195 were downregulated and that their common putative target gene Bcl-2 was upregulated in the RIPerc group. However, the differences in miR-21 and miR-24 expression, together with programmed cell death 4 (PDCD4), which is the target gene of miR-21, were not significant. These findings provide some insight into the role of miRs in the cardioprotective effects induced by RIPerc.

Ischemic reperfusion (I/R) injury is an unavoidable event during cardiac surgery and is associated with postoperative morbidity and mortality. Therapies for myocardial I/R injury are evolving and have been exhaustively reviewed recently^1^,^2^. Among those promising cardioprotective strategies, remote ischemic conditioning (RIC), which is triggered by applying several cycles of brief I/R in distant organs or limbs, has emerged as the most practicable and attractive one against myocardial I/R injury^3^. The cardioprotective effects of RIC have been demonstrated by increasing numbers of studies, including animal experiments and clinical trials^4^,^5^,^6^,^7^,^8^. In addition to cardioprotection, RIC is also beneficial for preventing organs other than the heart from I/R injury^9^,^10^,^11^.

A novel alternative approach to RIC, remote ischemic perconditioning (RIPerc) was first introduced by Schmidt *et al.* in 2007^12^ and is triggered by brief repetitive ischemic and reperfusion periods in remote organs or limbs during target organ ischemia. RIPerc differs from other types of RIC, including remote pre- and post-conditioning, in terms of intervention timing and has many beneficial features from a practical viewpoint. We previously demonstrated that RIPerc reduced myocardial injury in patients undergoing valve replacement surgery, as evidenced by a lower release of cardiac troponin I (cTnI)^13^, and that it contributed to the prevention of cardiac surgery-associated renal injury^14^. The cardioprotective effects of RIPerc have also been supported by many other studies^15^,^16^. However, the underlying mechanisms of RIPerc remain to be clarified and have been discussed in detail in recent reviews^16^,^17^.

MicroRNAs (miRs) are natural, non-coding, single-stranded small RNA molecules with approximately 20 nucleotides that regulate gene expression through mRNA degradation and translation repression. Recently, many miRs have been identified in I/R injury and apoptosis regulation^18^,^19^,^20^. Moreover, some of these miRs have been found to be involved in the mechanisms of ischemic conditioning. For example, Yin and colleagues demonstrated that miR-21 was upregulated by ischemic preconditioning and reduced apoptosis in myocardium by repressing the programmed cell death 4 (PDCD4) gene^21^. Duan *et al.* found that miR-1 and miR-21 expression differed between different conditioning groups, indicating that the miRs expression is distinctively regulated by different conditioning protocols^22^. Slagsvold *et al.* demonstrated that miR-133a, miR-133b and miR-1 increased in atrial myocardium during coronary artery bypass graft surgery (CABG), whereas remote ischemic preconditioning (RIPC) inhibited the increase of miR-1 expression^23^. However, information regarding miR expression change during cardiac surgery is limited, and which miRs are involved in the cardioprotective effects of RIPerc is unknown.

Because apoptosis is one of the leading causes of I/R injury, we hypothesized that RIPerc may reduce apoptosis in myocardium via regulating the expression of certain miRs. In the present study, we attempted to investigate whether RIPerc applied during cardiac surgery can exert its cardioprotective effect by inhibiting apoptosis in atrial myocardium and detect the expression changes of apoptosis-related miRs and their putative target genes.

## Methods

### Study design

Thirty patients with rheumatic valvular disease undergoing double valve (mitral and aortic valve) replacement surgery in our hospital were randomized into an RIPerc group (n = 15) and control (Con) group (n = 15) (See Fig. S1). Patients with atrial fibrillation (AF), coronary artery disease, previous heart surgery history, infective endocarditis, peripheral vascular diseases, diabetes, and hepatic, renal or pulmonary disease were excluded. Patients taking aspirin, corticosteroids, angiotensin-converting enzyme inhibitors, or statin were also excluded. A 12-cm-wide blood pressure cuff placed around the patient’s right thigh was used to trigger RIPerc as previously described^13^. After cross-clamping the aorta during surgery, the pressure cuff was immediately inflated to 600 mmHg to block blood flow to the lower limb according to the orthopedic standard for 5 minutes, followed by 5-minute deflation with 3 such cycles in the RIPerc group, whereas the cuff remained deflated in the control group. Patients, surgeons, perfusionists, ICU staff and lab technicians were blinded to the grouping of patients.

### Ethics

This study was approved by the Ethic Committee of Xiangya Hospital, Central-South University, China, and was performed in compliance with the Declaration of Helsinki. Written informed consent was obtained from patients prior to inclusion.

### Anesthesia and surgery

The anesthetic and surgical procedures were performed as previously described in detail^13^. Briefly, intravenous midazolam and vecuronium bromide were used for the induction of general anesthesia, which was maintained by intravenous fentanyl and propofol and intermittent inhalation of isoflurane. The surgeries were performed by the same surgeon and perfusion team. Median sternotomy, standard cardiopulmonary bypass and moderate hypothermia (28–31 °C) were used. An intermittent antegrade perfusion of 1:4 cold crystalloid-blood cardioplegia was administered for cardiac arrest. The mitral valve replacement was conducted *via* right atrial septa incision, and aortic valve replacement was conducted *via* aortic root transverse incision. Repair of the tricuspid valve with Kay’s technique^24^ was performed after removal of aortic cross-clamping if needed.

### Clinical data and blood samples for cTnI

All patients received standard postoperative care and were followed up for at least 30 days after surgery. Data on ventilation time, intensive care unit stay, X-ray, electrocardiography, echocardiography and major complications after surgery were collected. Serum cTnI concentrations were measured in blood samples taken from peripheral veins at different time points: preoperative (T0), 12 hours (T12), 24 hours (T24), and 48 hours (T48) after surgery, using enzyme-linked immunosorbent assays according to the supplier’s instructions (Jidan Biotechnology Co., Nanjing, China).

### Atrial myocardium harvest

During the valve replacement surgery, a small piece of right atrial appendage was excised prior to starting cardiopulmonary bypass and 5 minutes after removal of aortic cross-clamping. Blood on atrial myocardium tissues was rapidly rinsed off with ice-cold saline, and the tissues were then snap-frozen in liquid nitrogen and kept at −80 °C until further analyses were carried out.

### Apoptosis assay

Apoptosis in atrial myocardium samples was detected using the terminal deoxynucleotidyl transferase (TDT)-mediated dUTP nick-end labeling enzyme method (TUNEL). Briefly, sections were deparaffinized, permeabilized with proteinase K, and incubated with a TDT solution. Then, the sections were exposed to streptavidin-horseradish peroxidase solution and visualized by 3,3′-diaminobenzidine (DAB; Golden Bridge Biotechnology, Beijing, China). After washing, the sections were counterstained with hematoxylin. Nuclei from the *in situ* apoptotic cells were stained brown. The total number of cardiomyocytes and number of TUNEL-positive cardiomyocytes were manually counted in ten random fields for each section under a light microscope ((Olympus Optical, Tokyo, Japan). The apoptotic index (AI) was defined as the percentage of TUNEL-positive cardiomyocytes in the total number of cardiomyocytes.

### MicroRNA array chip assay

Total RNA was extracted using a TRIzol reagent kit (Invitrogen, Carlsbad, CA, USA) in accordance with the manufacturer’s instructions. The final concentration and purity were measured by an ultraviolet absorption assay at 260/280 nm.

RNA samples (n = 5 in each group) were labeled using a miRCURY™ Hy3™/Hy5™ Power labeling kit (Exiqon, Skelstedet, Vedbaek, Denmark) and hybridized with the miRCURY™ LNA Array Chip (v.14.0, Exiqon, Skelstedet, Vedbaek, Denmark). The hybridized signals were detected with an Axon GenePix 4000B microarray scanner. Images were acquired and analyzed using GenePix Pro 6.0 software. After background correction and normalization using the quantile method, at least a 1.5-fold change of microRNA expression with P < 0.05 was used as the inclusion criteria.

### Quantitative RT-PCR

Purified RNA samples were reverse transcribed into cDNA using a reverse-transcription kit (Promega Co., Madison, WI, USA). Two RT reactions for each sample were pooled and diluted with an equal amount of DNase/RNase free water. Real-time PCR was performed with 2 μL of diluted RT product in a Gene Amp PCR System (Applied Biosystems, Foster city, CA, USA) using iQ™ SYBR Green PCR Supermix (Bio-Rad, Hercules, CA, USA) according to the manufacturer’s instructions. Specific primers were designed with Primer 5 (PREMIER Biosoft International, Palo Alto, CA, USA). U6 was used as reference for miRs and GAPDH for apoptosis-related genes. The designed primers are listed in Table 1.

### Western blot (WB)

Myocardium tissues were homogenized in RIPA buffer (50 mM Tris HCl, pH 8, 150 mM NaCl, 1% NP-40, 0.5% sodium deoxycholate, 0.1% SDS). Protein concentration was determined using a Pierce BCA protein assay kit (Pierce, Rockford, IL, USA). After electrophoresis, proteins were transferred onto PVDF membrane. The membrane was blocked with 5% BSA and then incubated with primary antibodies overnight at 4 °C. After washing, the secondary antibody with HRP labeling was applied for 1 hour at room temperature. Then, the membrane was incubated with KC™ chemiluminesence reagents (Kangchen Biology Ltd., Shanghai, China) and exposed to X-ray. The final images were analyzed using ImageJ (NIH, USA). All primary and secondary antibodies were purchased from Kangchen Biology Ltd, Shanghai, China.

### Statistical analysis

Statistical analysis was performed with SPSS 17.0 software (SPSS Inc., Chicago, IL, USA). Data are presented as the mean ± standard deviation (SD) unless otherwise noted, and a Shapiro-Wilk test was used to test the normality of the data. Categorical data, including sex and incidence of postoperative AF, were compared with the Pearson *x*^2^ test and Fisher exact test. Comparison of normally distributed data, such as perioperative characteristics, between the two groups was performed using a student’s t-test. Data of non-normal distribution derived from miR assay, qRT-PCR, and WB were compared with a Mann-Whitney rank sum test, and the serum cTnI levels obtained at different time points were analyzed using a repeated-measures analysis of variance. P < 0.05 was considered significant.

## Results

Major complications and in-hospital mortality did not occur in this study. Perioperative patient characteristics and clinical parameters are summarized in Table 2. The two groups did not differ in preoperative characteristics, including age, weight, sex, NYHA classification and LVEF, or in other parameters, such as total surgery duration, cardiac ischemic duration, ventilation duration, ICU stay and hospital stay. Postoperatively, atrial fibrillation (AF) occurred in 2 patients in the RIPerc group and 4 patients in the control group, but the difference was not significant.

### Myocardial injury

**Figure 1:**
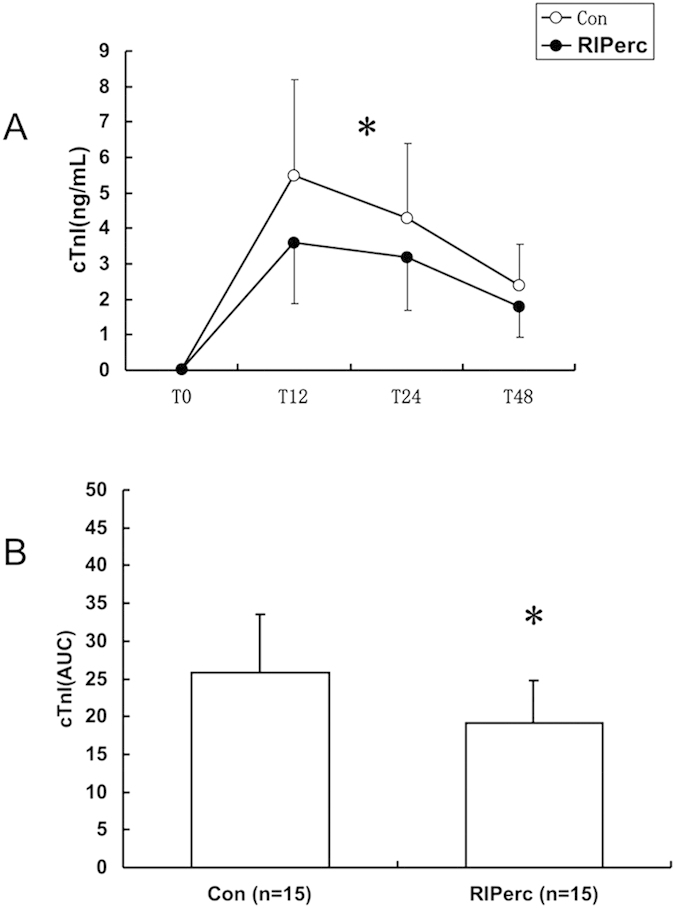
Postoperative release of serum cTnI. (**A**) Serum cTnI levels at different time points. (**B**) Total release of cTnI presented as the area under curve (AUC). T0, T12, T24 and T48 indicate preoperative and 12, 24 and 48 hours after surgery, respectively. * vs Control, P < 0.05.

Serum cTnI levels prior to surgery were comparable between groups and increased after surgery in both groups, peaking at 12 hours. However, serum cTnI levels at 12, 24 and 48 hours after surgery were lower in the RIPerc group compared with control, as well as the total release of cTnI expressed as the area under the curve (AUC) over the 48-hour postoperative period (19.11 ± 15.38 vs 25.8 ± 27.66, P = 0.04) (Fig. 1).

### Apoptotic indexes in atrial myocardium

**Figure 2:**
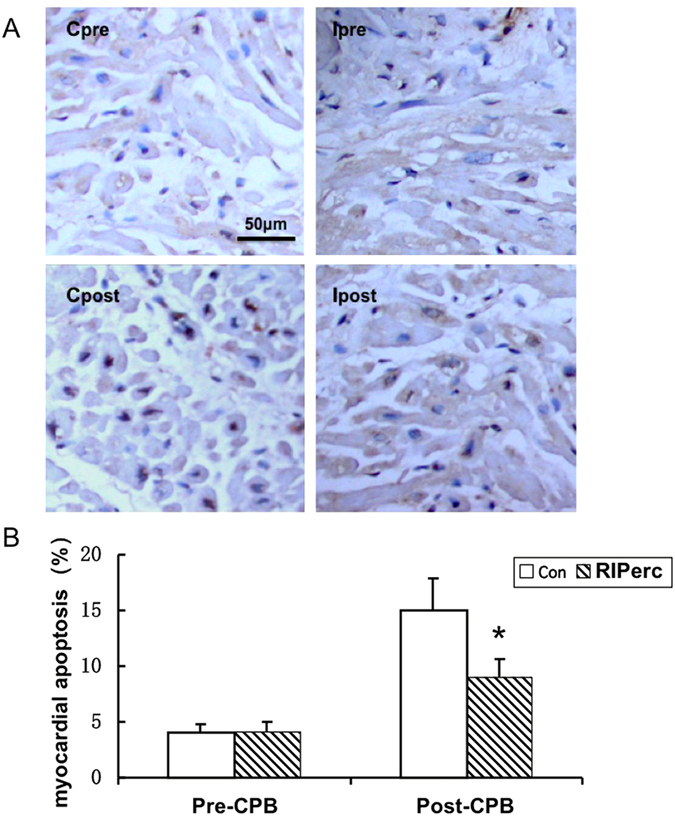
Apoptosis in atrial myocardium. (**A**) Representative images of apoptosis in both groups. Cpre and Ipre prior to aortic cross-clamping (ACC) in control and the intervention group (RIPerC); Cpost and Ipost after removal of ACC in control and the intervention group (RIPerC). Scale bar (50 μm) applies to all images. (**B**) Apoptosis index in atrial myocardium prior to and after cardiac ischemia in both groups. * vs Con, P < 0.05.

Prior to aortic cross-clamping, the AI of atrial myocardial samples in both groups were similar (4.2 ± 1.2% vs 4.7 ± 1.5%, p = 0.65). It increased remarkably during cardiac ischemia. However, the AI in the RIPerc group (n = 15) was remarkably lower than in the control group (n = 15) after removal of aortic cross-clamping (8.7 ± 2.7% vs 16.0 ± 5.5%, p = 0.023) (Fig. 2).

### MiR expression

**Figure 3:**
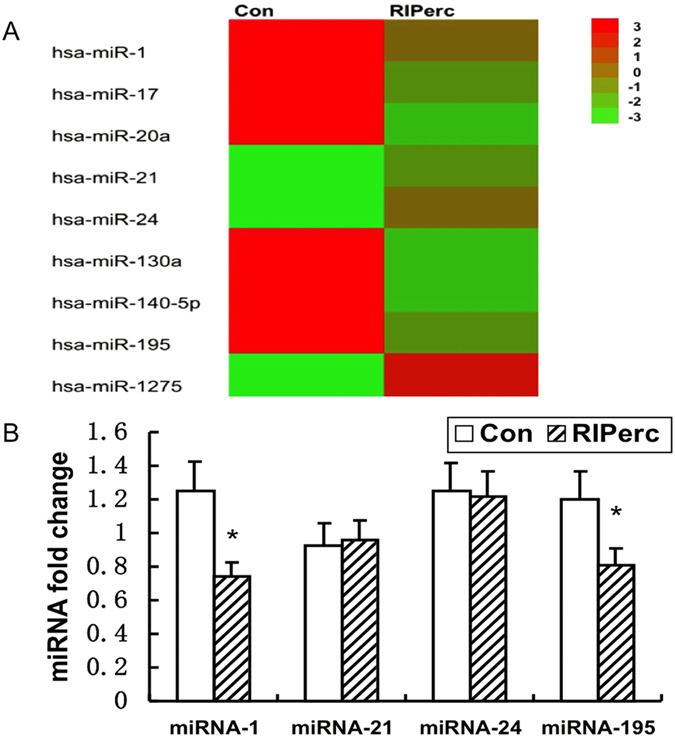
Expression change of microRNAs (miRs) in atrial myocardium during valve replacement surgery. (**A**) Nine miRs exhibited different expression changes between control (Con) and the RIPerC group. (**B**) Expression change of miR-1, miR-21, miR-24 and miR-195 in both groups. * vs Control, P < 0.05.

In the present study, with the miRNAs array chip technique we found that 17 human miRs showed significant expression change in atrial myocardium after ischemia during cardiac surgery, including up-regulation of miR-1, miR-17, miR-19b, miR-20a, miR-33a, miR-130a, miR-140-5p, miR-195, miR-301a, miR-324-5p, miR-377, miR-491-5p, and miR-657, and down-regulation of miR-21, miR-24, miR-1275, and miR-1973. Furthermore, by comparison between the RIPerc group (n = 5) and the control group (n = 5), there were significant differences in 9 miRs of the 17 miRs, with increased expression of miR-21, miR-24, and miR-1275 as well as decreased expression of miR-1, miR-17, miR-20a, miR-130a, miR-140-5p, and miR-195 in the RIPerc group (Fig. 3A).

### Quantitative RT-PCR for miRs

Because apoptosis was the focus of this study, 4 miRs, including miR-1, miR-21, miR-24, and miR-195, which have been reported to be involved in apoptosis regulation, were selected for further study. Using quantitative RT-PCR, our data indicated that miR-1 and miR-195 were significantly downregulated in the RIPerc group (n = 15) compared with control (n = 15), which was in accordance with miRNAs array chip results. However, the increased miR-21 and miR-24 expression described above was not confirmed because no intergroup difference was observed in the RT-PCR assay (Fig. 3B).

### Expression of apoptosis-associated genes

**Figure 4:**
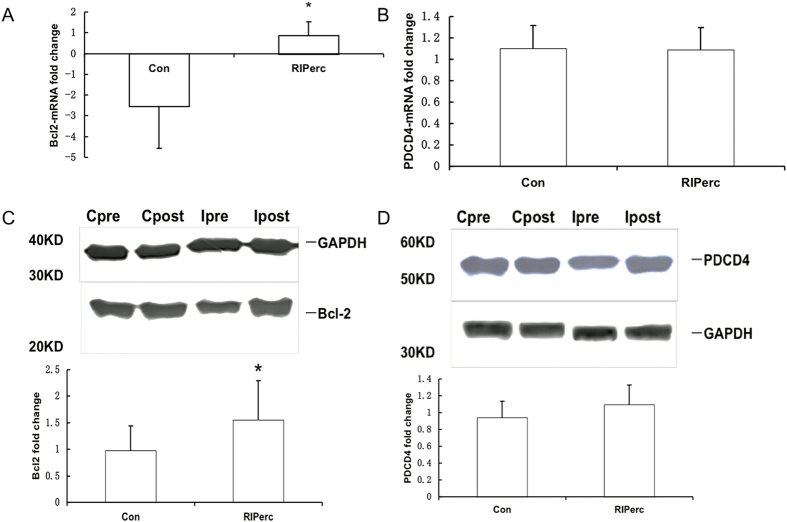
Expression of miR-targeted genes. (**A**) Expression change of Bcl-2 mRNA. (**B**) Expression change of PDCD4 mRNA. (**C**) Expression change of Bcl-2 protein. (**D**) Expression change of PDCD4 protein. Cpre and Cpost prior to and after cardiac ischemia in control; Ipre and Ipost prior to and after cardiac ischemia in the RIPerc group. * vs Control, P < 0.05.

Expression of B-cell lymphoma 2 (Bcl-2), which is the putative target gene of miR-1 and miR-195^25^,^26^, and programed cell death 4 (PDCD4), which is the putative target gene of miR-21^27^, was studied at the mRNA and protein levels using quantitative RT-PCR and Western blotting. Bcl-2 and PDCD4 expression levels prior to ischemia were similar between groups. After ischemia, Bcl-2 expression was significantly increased at both the mRNA and protein levels in the RIPerc group (n = 15) compared with control (n = 15), whereas no difference was observed in PDCD4 expression between groups (Fig. 4).

## Discussion

Our primary findings were that RIPerc administered during the valve replacement surgery attenuated myocardial injury and reduced apoptosis in right atrial myocardium. More importantly, we demonstrated that miR-1 and miR-195 expression was decreased in the RIPerc group but their common target gene, Bcl-2, was increased in atrial myocardium during ischemia. Additionally, we found increased miR-21 and miR-24 expression induced by RIPerc with a miRNAs array chip; however, these results were not confirmed by quantitative RT-PCR.

The mechanisms of I/R injury have been extensively reviewed in the literature but remain to be elucidated^28^,^29^. I/R injury is an inevitable event during cardiac surgery, and preventing cell death by inhibiting apoptosis is an effective strategy to antagonize I/R injury in myocardium because cardiomyocytes are terminally differentiated and have little potential for regeneration^30^. Our results revealed that apoptosis in right atrial myocardium increased during cardiac ischemia, and RIPerc administered during surgery was beneficial for reducing apoptosis in atrial myocardium, which partly explain its cardiopretective effects, as evidenced by the decreased release of serum cTnI. However, the underlying mechanisms of anti-apoptotic effect of RIPerc were unclear.

Growing evidence has shown that miRs play a central role in I/R injury and apoptosis regulation. Because miRs have great potential as therapeutic targets^31^, we wanted to investigate whether miR-related anti-apoptotic pathways are involved in the cardioprotective effect of RIPerc. With the miRNA array chip, we found expression changes in 17 miRs during cardiac ischemia, and most of these miRs are associated with oxidation stress, inflammation, ion channels and apoptosis. Through comparisons with the control group, 9 of the 17 miRs exhibited different expression changes in the RIPerc group. Of these 9 miRs, 4 miRs, including miR-1, miR-21, miR-24, and miR-195, which are reported to be involved in regulating apoptosis, were selected for further study. The decreased miR-1 and miR-195 expression in the RIPerc group was confirmed by quantitative RT-PCR, whereas the increased miR-21 and miR-24 expression was not.

miR-1 is abundantly expressed in striated muscle, including myocardium. Our study demonstrated that miR-1 expression in atrial myocardium was remarkably increased after cardiac ischemia, which was in agreement with previous studies^32^,^33^. Tang and colleagues demonstrated miR-1 overexpression facilitated H_2_O_2_-induced apoptosis in cardiomyocytes by post-transcriptional repression of Bcl-2^25^. Interestingly, Slagsvold *et al.*^23^ showed that RIPC inhibited the increase of miR-1 expression in atrial myocardium during CABG surgery, which was in line with our results, although different intervention approaches were employed in different surgeries, indicating that miR-1 may be the common target of RIPerc and RIPC. The authors also noted a lower incidence of postoperative AF in the RIPC group, which was not observed here.

miR-195 also exhibited a different expression profile between the groups and has been reported to be involved in cell cycle and apoptosis regulation^34^. However, the significance of miR-195 expression change and whether miR-195 regulates apoptosis induced by I/R injury remain unknown. Zhu *et al.*^35^ revealed that miR-195 promoted palmitate-induced apoptosis in cardiomyocytes by down-regulating sirt-1, and Chen *et al.*^26^ demonstrated that miR-195 promoted apoptosis in mouse podocytes by targeting Bcl-2.

Taken together, Bcl-2 appears to be one of the common targets of miR-1 and miR-195 to regulate apoptosis. Bcl-2 prevents cell from apoptosis by forming heterodimers with other pro-apoptotic members in its family^36^. In the present study, using quantitative RT-PCR and WB, our data indicated that Bcl-2 was up-regulated in right atrial myocardium in the RIPerc group at both the mRNA and protein levels. Therefore, we hypothesized that RIPerc inhibits the increase in miR-1 and miR-195 expression in atrial myocardium during ischemia, resulting in increased Bcl-2 expression, which attenuates apoptosis in myocardium. The cause-effect relationship in our hypothesis was not well established or fully supported by our findings. Further mechanistic studies are thus needed.

Of note, the functions of miR-1 and miR-195 are not limited to apoptosis regulation in myocardium. Multiple lines of evidence have shown that miR-1 is involved in cardiac hypertrophy, arrhythmia and mitochondrial function^23^,^37^. miR-195 was found to act as a tumor suppressor by targeting multiple signaling pathways in different tumors^38^,^39^. Therefore, the significance of miR-1 and miR-195 expression changes during cardiac surgery remains unresolved.

Both miR-21 and miR-24 have been found to exert anti-apoptotic effects in myocardium^27^,^40^. As described above, miR-21 may regulate apoptosis by targeting PDCD4. Yang *et al.* reported that miR-21 protects cardiocytes against apoptosis via phosphatase and tensin homolog/Akt-dependent mechanisms^41^. Wang *et al.* showed that the miR-24 mediated anti-apoptotic effects occurred through the regulation of intrinsic apoptosis pathways in mouse cardiomyocytes^42^. Here, miR-21 and miR-24 expression in atrial myocardium was increased after ischemia, but the difference between the two groups via miRNA array chip assay was not replicated in the quantitative RT-PCR assay. Additionally, the expression of PDCD4 at the mRNA and protein levels was not different between the RIPerc group and control. However, given the limited sample size in the present study, further studies are needed to exclude the possibility of a false negative result.

In conclusion, our study demonstrated that RIPerc applied during cardiac surgery exerted cardioprotective effects, which can be partly explained by its ability to inhibit apoptosis in myocardium. Further, RIPerc altered the expression profile of apoptosis-related miRs, which may help better understand the underlying mechanisms of RIPerc. Because miRs have great potential as therapeutic targets, our findings that miR-1 and miR-195 were downregulated by RIPerc may contribute to the exploration of novel therapeutic strategies to prevent I/R injury in the heart.

The limitations of this study are discussed as follows. First, several anesthetic agents, with preconditioning effects, including isoflurane, propofol and opioids^43^, were used in all patients in this study. Therefore, these anesthetic agents may have interfered with our results, especially when comparing the miR expression profile between the RIPerc group and control. Second, the tissue samples were only taken from the right atrium due to ethical issues. Given the difference between atrial and ventricular myocardium, our results in atrial myocardium cannot be easily translated to ventricular myocardium. However, because apoptosis is a very common mechanism of I/R injury in different cell types and is regulated by similar patterns, our results at least provide some insight into how RIPerc protects myocardium against apoptosis via miR-related pathways. Third, the tissue samples from right atrium compose cell types other than cardiomyoctes, such as fibroblast, smooth muscle cell, and endotheliocyte, which may have caused some interference in our results. Lastly, as proposed by Baars *et al.* in their study^44^, miRs may indeed play some roles in myocardial I/R injury, but the causal link between miRs and the cardioprotective effects of RIPerc is far from being established.

## Additional Information

**How to cite this article**: Hu, Q. *et al.* Apoptosis-related microRNA changes in the right atrium induced by remote ischemic perconditioning during valve replacement surgery. *Sci. Rep.* **6**, 18959; doi: 10.1038/srep18959 (2016).

## Supplementary Material

Supplementary Information

## Figures and Tables

**Table 1:** List of the designed primers.

miR or mRNA	(RT) Primers
U6	5′CGCTTCACGAATTTGCGTGTCAT3′
hsa-miR-1	5′GTCGTATCCAGTGCGTGTCGTGGAGT
	CGGCAATTGCACTGGATACGACATACATA3′
hsa-miR-21	5′GTCGTATCCAGTGCGTGTCGTGGAGTCGGCA
	ATTGCACTGGATACGACTCAACAT3′
hsa-miR-24	5′GTCGTATCCAGTGCGTGTCGTGGAGTCGGC
	AATTGCACTGGATACGACCTGTTC3′
hsa-miR-195	5′GTCGTATCCAGTGCGTGTCGTGGAGTCG
	GCAATTGCACTGGATACGACGCCAATA3′
BCL-2	F:5′ACTTCGCCGAGATGTCCAG3′
PDCD4	R:5′AATCCTCCCCCAGTTCACC3′
GAPDH	F:5′GGATGAAAGGGCATTTGAGAA3′
	R:5′CCCCTCCAATGCTAAGGATACT3′
	F:5′GGGAAACTGTGGCGTGAT3′
	R:5′GAGTGGGTGTCGCTGTTGA3′

**Table 2:** Patient characteristics and perioperative parameters.

		RIPerc	CON.	P
sex	Male	6	5	0.685
	Female	9	10	
Weight (kg)		53.4 ± 8.9	51.0 ± 5.8	0.348
Age (years)		43.2 ± 8.0	45.7 ± 7.2	0.385
NYHA class		2.5 ± 0.63	2.7 ± 0.59	0.353
LVEF		55.8 ± 10.4	53.8 ± 9.0	0.265
CPB (minutes)		101.6 ± 24.5	91.8 ± 14.6	0.132
ACC (minutes)TVP		78.5 ± 22.510/15	68.3 ± 11.68/15	0.1250.709
Total (minutes)		204 ± 43	190 ± 31	0.173
Ventilation (hours)		8.6 ± 3.7	10.7 ± 5.3	0.215
ICU stay (hours)		30.0 ± 13.5	29.7 ± 18.4	0.915
Hospital stay (days)Postoperative AF		9.1 ± 3.52/15	8.7 ± 2.94/15	0.7450.363

LVEF: left ventricular ejecting fraction; CPB: cardiopulmonary bypass; ACC: aortic cross-clamping; TVP: tricuspid valvuloplasty; ICU: intensive care unit; AF: atrial fibrillation.
