# Supplementary material for: Apoptosis-related microRNA changes in the right atrium induced by remote ischemic perconditioning during valve replacement surgery
Source: Sci Rep. 2016 Jan 7;6:18959. doi: 10.1038/srep18959 (PMC4704063; doi:10.1038/srep18959)
Supplement: Supplementary Information [file srep18959-s1.doc]

**Apoptosis-related microRNA change in the right atrium induced by remote ischemic perconditioning during valve replacement surgery**

Qinghua Hu^1^, Wanjun Luo^1，*^ , Lingjin Huang^1^ , Rimao Huang^1^ , Ri Chen^1^

**Supplement material**

**Assessed for eligibility (n=30)**

**Excluded (n=0)**

**Randomized (n=30)**

**DVR (n=30)**

**CON**

**RIPerc**

**n=15**

**n=15**

Figure S1. Diagram of inclusion process and grouping


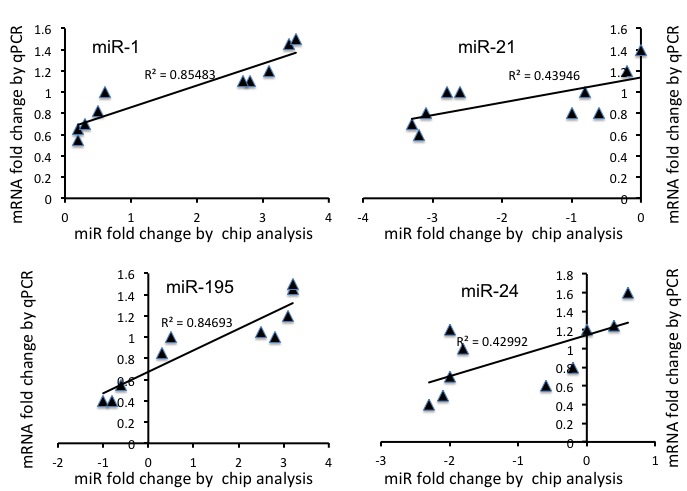


Figure S2. Pairwise correlation plot of miRNAs fold change detected by miRs chip and qPCR.
